# Urinary Arsenic Metabolites in Children and Adults Exposed to Arsenic in Drinking Water in Inner Mongolia, China

**DOI:** 10.1289/ehp.9271

**Published:** 2007-01-09

**Authors:** Guifan Sun, Yuanyuan Xu, Xin Li, Yaping Jin, Bing Li, Xiance Sun

**Affiliations:** Department of Environmental and Occupational Health, College of Public Health, China Medical University, Shenyang, Liaoning, People’s Republic of China

**Keywords:** arsenic, children, drinking water, metabolism, methylation

## Abstract

**Background:**

We report the concentrations and distributions of urinary arsenic (As) metabolites in 233 residents exposed to 20, 90, or 160 μg/L inorganic arsenic (iAs) in drinking water from three villages in Hohhot, Inner Mongolia, China, that formed one control and two exposed groups.

**Methods:**

We used hydride generation-atomic absorption spectrometry (HGAAS) to determine iAs, monomethylarsonic acid (MMA), and dimethylarsinic acid (DMA).

**Results:**

The concentrations of each urinary As species in the two exposed groups were significantly higher than in the control group for both children and adults. Both children and adults in exposed groups had higher percent iAs and MMA and lower percent DMA, and low primary and secondary methylation indices (PMI and SMI, respectively) than those in the control group. However, children showed significant increases in percent DMA and the SMI as well as decreases in the percent MMA when the iAs exposure level increased from 90 to 160 μg/L. In addition, children in the two exposed groups showed lower percent MMA but higher percent DMA and higher SMI than adults in the same exposed group. No significant differences in As metabolite concentrations and distributions were found between males and females in each group. A significant correlation was also found in the SMI between 11 pairs of children and their mothers from the 160-μg/L–exposed group.

**Conclusions:**

Children had higher a capacity for secondary methylation of As than adults when exposed to the same concentrations of iAs in drinking water. Exposure to As may increase the capacity for methylation in children to some extent.

Arsenic (As), a ubiquitous element in nature (Cullen and Reimer 1989), has been identified as a human carcinogen by the International Agency for Research on Cancer (IARC 1987). Chronic exposure to drinking water containing high levels of inorganic arsenic (iAs) is associated with manifestations of various skin diseases (Tondel et al. 1999), diabetes (Bates et al. 1992; Tseng et al. 2000), cardiovascular disease (Engel et al. 1994), and cancers of several organs (Bates et al. 1992; Chen et al. 2005).

Recent development of analytical methods for As speciation has resulted in a large number of publications on As metabolism in humans. Until now, two important theories concerning As biotransformation attracted much attention of researchers in this field. One theory is that the classic metabolic pathway of As in mammals consists mainly of two reduction steps and two methylation steps as follows: iAs^V^ → iAs^III^ → monomethylarsonic acid (MMA^V^) → MMA^III^ → dimethylarsinic acid (DMA^V^) (Aposhian et al. 2004). The other theory is that the new As pathway is based on the formation of As–glutathione complexes (Hayakawa et al. 2005). These complexes cannot be detected in human urine because they are degraded by γ-glutamyltransferase (GGT) in the microvilli of the proximal tubules in the kidney. The products of the degradation may be transformed into iAs, MMA, or DMA (Kala et al. 2004). However, both theories suggest the two methylation steps are involved in the metabolism of As. If these theories are correct, then methylation is key to understanding the biotransformation of As. In addition, trivalent forms of As are more toxic than the pentavalent forms of As, with MMA^III^ being the most toxic As metabolite (Bing et al. 2001; Nesnow et al. 2002; Styblo et al. 2000). Cytotoxicity assays revealed the following order of toxicity of the arsenicals: MMA^III^ > iAs^III^ > iAs^V^ > MMA^V^ = DMA^V^ (Petrick et al. 2000). Consequently, the traditional belief that biomethylation was a detoxification pathway of iAs has been questioned (Kurttio et al. 1998; Styblo et al. 2002). The new understanding of As metabolism creates interesting challenges in evaluating health effects of in populations exposed to iAs because of large variations in the factors that affect metabolism, such as nutrition (Gamble et al. 2005), genetics (Chung et al. 2002), sex (Shraim et al. 2003; Tseng et al. 2005), and age (Chowdhury 2003; Tseng et al. 2005).

Exposure to As in drinking water at concentrations as low as 50 μg/L has been shown to adversely affect children’s intellectual function in Bangladesh (Wasserman et al. 2004), but little is known about the potential differences, such as concentrations or distribution of urinary As metabolites in children and adults. Here, we report the results of a cross-sectional study we conducted in Inner Mongolia, China, on the concentrations and the distribution of urinary As metabolites (iAs, MMA, and DMA) in children and adults exposed to 20, 90, or 160 μg/L As in groundwater used for drinking. In addition, comparing urinary As concentrations and the distribution of As species, we also apply two indices, the primary methylation index [PMI = (MMA + DMA)/total As (TAs)] and the secondary methylation index [SMI = DMA/(MMA + DMA)], to evaluate As methylation ability. PMI calculated as MMA/iAs and SMI calculated as DMA/MMA were previously developed for As methylation ability (Chen et al. 2003a, 2003b; Tseng et al. 2005). Our new methods for calculating PMI and SMI are more logical. According to the pathway of As metabolism, secondary methylation can only proceed on the basis of primary methylation; consequently, parts of the As products resulting from primary methylation are further methylated. Therefore, to evaluate the primary methylation ability, not only the primary but also the secondary methylation products should be considered. PMI and SMI show differences in As metabolism between children and adults but not between males and females. Finally, we report results of urinary As metabolites in 11 pairs of children and their mothers to illustrate the possible role of genetics in As metabolism.

## Methods

### Study population

We recruited 233 subjects from three villages—Tianjiaying, Koukenban, and Naimoban near Hohhot, Inner Mongolia, China. The control group with 36 individuals (mean age, 22.6 years) resided in Tianjiaying village on the outskirts of Hohhot, where the deep tube-well water with an iAs concentration of 20 μg/L was provided by centralized waterworks. Currently, the drinking water standard in China for iAs is 50 μg/L (Ministry of Health 2006). In the other two villages, a centralized tap water system was established in 1998 to eliminate fluoride from tube well water. Unfortunately, the new tap water system contained concentrations of iAs exceeding 50 μg/L and concentrations in Naimoban and Koukenban were 90 and 160 μg/L, respectively. The number of villagers participating in this study was 97 from Naimoban (mean age, 26.8 years) and 100 from Koukenban (mean age, 25.4 years). In addition, we also recruited 11 pairs of children (age range, 2–5 years) and their mothers (age range, 25–31 years) from Koukenban.

We have complied with the Declaration of Helsinki Ethical Principles for Medical Research Involving Human Subjects (World Medical Association (World Medical Association 1989). All 233 recruited villagers gave informed consent before participating. Data on health status, cigarette smoking, dietary habits, history of chronic disease, family members, place of birth, race, education, economic conditions, and reproductive conditions were obtained by questionnaire. The distribution of age was similar for males and females in each group. Trained medical doctors conducted detailed physical examinations according to the Diagnosis Standards on Arsenicosis of China (WS/T211-2001; Ministry of Health 2001) to identify cases of arsenicosis. Previously, there had been no cases of arsenicosis identified in the villages in this study. The subjects were generally healthy and had no history of other chronic diseases.

### Sample collection

Water samples were collected from the delivery ports of the deep wells and the waterworks and stored in 15-mL polypropylene conic tubes (Becton Dickinson Labware, Franklin Lakes, NJ, USA) without acidification and kept on ice. Our previous studies have demonstrated that iAs in water samples without acidification from these villages was stable when stored at 4°C within 1 month of sampling (unpublished data). Sampling took place three times in three different seasons. Samples of first-morning void urine (5 mL) were obtained in 15-mL polypropylene tubes (Sarstedt, Tokyo, Japan) and kept on ice. Within 8 hr, the urine samples were taken to the Centre of Disease Control in Hohhot and stored frozen at −20°C for 5 days. Typically, a dozen urine samples and water samples, kept on dry ice, were sent to the Laboratory of Arsenic Analysis in China Medical University (Shenyang, Liaoning province, China). Water samples were analyzed upon arrival; urine samples were stored at −80°C and analyzed within 3 months.

### Reagents and standards

Arsenate (Na_3_AsO_4_ • 12H_2_O), arsenite (NaAsO_2_), HCl, NaOH, and NaBH_4_ were purchased from the Shanghai Chemical Co. (Shanghai, China). All reagents used in this study are analytical grade (the highest grade commercially available in China) and As free (< 0.01 mg/L). We used a mixed As standard of 1,000 mg/L MMA, DMA, and trimethylarsonic acid (TMA) (Tri Chemical Laboratories Inc., Yamanashi, Japan). We acquired an iAs standard of 1,000 mg/L from the National Center for Standard Reference Materials (Beijing, China). Standard reference material of freeze-dried urine (SRM 2670) for toxic metals was obtained from the U.S. National Institute of Standards and Technology (NIST; Gaithersburg, MD, USA).

### Determination of As metabolites

We determined As species (iAs, MMA, DMA, and TMA) in urine using atomic absorption spectrophotometer (AA-6800) with an As speciation pretreatment system (ASA-2SP; Shimadzu Co., Kyoto, Japan). Speciation analysis was based on the well-established hydride generation of volatile arsines, followed by cryogenic separation in liquid nitrogen. The limit of detection of 1 ng ± < 5% for each of the four As species was determined using hydride generation-atomic absorption spectrometry (HGAAS). Briefly, 1 mL urine that had been stored at −80°C was thawed at room temperature and digested with 2 N–NaOH solutions at 100°C for 3 hr in a 15-mL polymethylpentene test tube (Sarstedt), followed by dilution with Milli-Q water (Millipore, Yonezawa, Japan). This digestion procedure did not alter the distribution of iAs or methylated arsenicals (Yamauchi and Yamamura. 1984). The absorbance of As in the digested urine samples was determined at 193.7 nm. Quality control for As determinations included the analysis of SRM2670. The NIST-certified concentration values for As were 480 ± 100 μg/L. The values measured in our laboratory were 474 ± 20 μg/L. The reliability of As species separation was evaluated by the analytical recoveries of added As species. Spiking urine samples with 10 μg/L of iAs, MMA, DMA, and TMA resulted in recoveries of 81–92%, 88–98%, 89–103%, and 80–95% for iAs, MMA, DMA, and TMA, respectively. Because no TMA was detected in the urine samples of any subject, we report the TAs concentrations by summing the concentrations of iAs, MMA, and DMA. Under our analytical conditions, we could not differentiate the trivalent forms from the pentavalent forms of As.

### Creatinine in urine

Urinary creatinine was measured by Jaffe reaction with a commercial kit (Jiancheng Biological Institute of Nanjing, Nanjing, China). Concentrations of As species and TAs were normalized to creatinine concentrations to correct for urine dilution (Valenzuela et al. 2005).

### Statistical analysis

Data analysis was carried out using SPSS software (version 11.5, SPSS Inc., Chicago, IL, USA). The concentrations of As species, as well as PMI and SMI, were first log-transformed. Analysis of log-transformed data was performed using one-way analysis of variance (ANOVA) and the Student-Newman-Keuls (SNK) post hoc test. We used the paired *t*-test to compare child–mother differences. All values were transformed back to the arithmetic scale for reporting purposes.

## Results

### Distribution of urinary As metabolites between children and adults

Both children and adults had increased concentrations of each As metabolite and the TAs, as the concentration of iAs in drinking water increased from 20 μg/L (control) to 90 and 160 μg/L (Table 1). The urinary TAs of the 90- and 160-μg/L–exposed groups were, respectively, 7.8 and 15.6 times that of the 20-μg/L–exposed group in children and 26.3 and 68.4 times that in adults (*p* < 0.05; Table 1). In addition, urinary TAs of children and adults in the 160-μg/L–exposed group were, respectively, 1.88 and 2.54 times that in the 90-μg/L–exposed group (*p* < 0.05; Table 1).

Concentrations of all As metabolites were higher in children than in adults in the 20-μg/L–exposed control group and in the 90-μg/L–exposed group (*p* < 0.05). However, concentrations of all As metabolites were lower in children than in adults in the 160-μg/L–exposed group (*p* < 0.05). In the 160-μg/L–exposed group, five adults had extremely high concentrations of all urinary As metabolites, about 5–10 times the average, for reasons that we do not yet understand. If we exclude the five adults, the geometric means (GMs) for iAs, MMA, DMA, and TAs (*n* = 51) would be lower—75, 71, 332, and 494 μg/g creatinine, respectively. GMs would also be lower than those of the children in the same group but without statistical significance. In the 11 child–mother pairs also from the 160-μg/L–exposed group, concentrations of all As metabolites were higher for children than for mothers (*p* < 0.01 except for MMA; Table 2).

The distribution of As metabolites among children and adults in the two exposed groups followed the same trend compared with the control group, with a decrease of percent DMA and an increase of percent iAs and MMA. Between the two exposed groups, percent iAs and MMA were decreased, whereas percent DMA was increased in children in the 160-μg/L–exposed group compared with the 90-μg/L–exposed group.

When exposed to the same concentration of iAs in drinking water, children generally had a higher percent DMA and a lower percent MMA than adults in the two exposed groups, which is consistent with the 11 child–mother pairs in the 160-μg/L–exposed group (Table 2).

### Distribution of urinary As metabolites between male and female

For the 90- and 160-μg/L–exposed groups, there were no significant differences in concentrations or percentages of any of the As metabolites between the male and female participants when they were exposed to the same concentration of iAs in drinking water (Table 3).

### PMI and SMI

**Figure 1:**
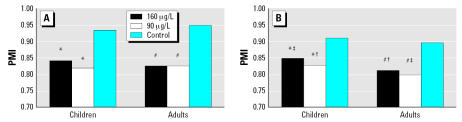
(*A*) Comparison of PMI in children and adults exposed to different levels of As in drinking water. Data are shown as geometric mean. (*B*) Comparison of SMI in children and adults exposed to different levels of As. Data are shown as geometric mean. Statistical significance: *p < 0.05, compared with children’s control group; ^#^*p* < 0.05, compared with adult control group; ^†^*p* < 0.05, compared with children in 160-μg/L–exposed group; ^‡^*p* < 0.05, compared with children in 90-μg/L–exposed group.

To facilitate the comparison of As metabolism between children and adults, we also calculated PMI [(MMA + DMA)/TAs] and SMI [DMA/(MMA + DMA)]. Both PMI and SMI values in adults and children significantly decreased in the 90-and 160-μg/L–exposed groups compared with the control group, but the decrease in SMI values occurred more in children than in adults (Figure 1A, B). In the control group, we found no significant differences in PMI and SMI values between children and adults (Figure 1B). However, for both the 90- and 160-μg/L–exposed groups, SMI values (Figure 1B) were significantly higher in children than in adults (*p* < 0.05). SMI values were also significantly higher in children in the 160-μg/L–exposed group than that in children in the 90-μg/L–exposed group (*p* < 0.05), whereas the difference in adults was not as large (Figure 1B). The slightly higher PMI values in children in the 160-μg/L–exposed group (Figure 1A) were not significantly different from that of adults in the same group. These results suggest that children’s secondary methylation ability is greater and more sensitive to As exposure than adults.

Children’s SMI values were higher than those of their mothers in the 11 child–mother pairs of in the 160-μg/L–exposed group (Table 4), which is consistent with the find-ings from the child–adult comparison. However, this difference was not significant because of the small sample size (Table 4). We did find an encouraging correlation of SMI values between children and their mothers (*r* = 0.655; *p* < 0.05). PMI values of children and mothers were comparable, although again without statistical significance, and there was no correlation between PMI values of children and their mothers (Table 4).

## Discussion

### Age differences in As excretion and metabolism

Higher levels of urinary As metabolites in children than in adults from both the 90-μg/L–exposed and control groups suggest that children’s ability to metabolize and excrete iAs was greater than in adults when exposed to the same concentrations of As (Table 1). Children’s absolute excretion of As in urine was less than that of adults in the 160-μg/L–exposed group when five adults with high levels of urinary As were included in the comparison. However, when adjusted for body weight, the concentration of urinary As species excreted in children was 1.5–1.9 times that of adults. After we excluded the five adults with unusually high levels of urinary As species and adjusted for body weight, excretion in children increased 2.2–2.8 times that in adults. These results indicate that adults might accumulate more As in their bodies than children do, based on the assumption that children and adults consume the same amount of As. A consistent feature of our data is that the percent DMA and SMI values in children in the two exposed groups were higher, whereas the percent MMA was lower than that in adults, suggesting that the second methylation step is higher in children, whereas the primary methylation step is not. The percent of urinary As species and the PMI and SMI values in children and adults in the control group were similar, which suggests that when the level of As in drinking water was low, methylation ability in both were comparable. Previous studies also found total urinary As excretion adjusted for body weight and the secondary methylation ability of iAs were higher in children than in adults (Chowdhury et al. 2003; Chung et al. 2002).

We do not know whether the better secondary methylation ability of children can explain why they appear to be less affected clinically by As exposure than adults exposed to the same concentration of As in drinking water (Chowdhury et al. 2003). However, this would be consistent with previous findings that the more cytotoxic MMA^III^ decreased when the level of DMA increased, as would be expected in the As metabolism pathway (Cullen et al. 1984; Styblo et al. 2002; Vahter 2002). That MMA is a a more toxic As metabolite than DMA is supported by the fact that higher levels and greater proportions of MMA have been found in individuals with arsenicosis than those in the control group who had no symptoms but who had been exposed to the same concentration of As (Calderon et al. 1999; Valenzuela et al. 2005).

In children, the secondary methylation ability was not only greater but also more sensitive to changes in the concentrations of As to which they were exposed. In exposed groups, both children and adults had lower As methylation ability, as indicated by much lower PMI and SMI values compared with the control group (Figures 1A, B). Del Razo et al. (1997) in a study of 35 individuals from Santa Ana, Mexico, where drinking water had an As concentration of 415 μg/L, observed that exposure to high levels of As could decrease the body’s ability to methylate As. However, a subtle increase of percent DMA (Table 1) and SMI values (Figure 1B) with a decrease in percent iAs and MMA (Table 1), which is significant for children, were observed in the 160-μg/L–exposed group compared with the 90-μg/L–exposed group. This observation indicates the induction of As methylation with increasing iAs exposure in children, and is consistent with the study of Concha et al. (1998). Although additional studies are needed to confirm this finding, the implication is that increasing iAs exposure does not always decrease children’s As methylation ability, rather it may induce As methylation ability within a certain range of iAs exposure concentrations.

### Gender and As metabolism

Comparison of urinary As between male and female participants indicates that the concentrations and distribution of As species do not vary by gender (Table 3). The primary and secondary methylation abilities of As were not different between men and women when they were exposed to the same levels of As. In Taiwan a study of 479 people exposed to As (700–930 μg/L) in drinking water from artesian wells found that women had a greater ability to methylate As than men (Tseng et al. 2005). Methylation of As was more efficient in women than in men in three populations studied: Mexico (*n* = 292), China (*n* = 37), and Chile (*n* = 21) (Loffredo et al. 2005). We cannot explain the apparent lack of difference in women and men in Inner Mongolia except to note that our study groups were exposed to lower concentrations of iAs. We cannot exclude the possible contribution of genetic differences in the study group.

### Genetics and As metabolism

The orders of proportions of urinary As metabolites was as follows: percent DMA > percent iAs > percent MMA for both of the exposed groups, and percent DMA >> percent MMA > percent iAs for the control group. This difference may be because of exposure to a measurable concentration of As in drinking water. The order of urinary As species concentrations in a population (*n* = 35) exposed to 396–470 μg/L iAs in drinking water from a town in Mexico was also percent DMA > percent iAs > percent MMA (Del Razo et al. 1997). Similar to most population groups studied to date, our two exposed groups have on average 10–30% iAs, 10–20% MMA, and 60–70% DMA in urine (Vahter 2000), but there was a considerable interindividual variation in the proportions of urinary As metabolite.

Could this variation be attributed to the genetic polymorphisms in As methyltransferase or MMA^V^ reductase (Concha et al. 2002; Karagas et al. 2004; Thomas et al. 2004)? Based on the variability of the urinary As metabolic profile in humans (Loffredo et al. 2003; Meza et al. 2004; Vahter 2000) and the differences in interspecies and intraspecies in animal models (Styblo et al. 1999; Thomas et al. 2001), genetic determinants are hypothesized to play an important role in As metabolism. A stronger correlation in As methylation-related phenotypes found among siblings than among genetically unrelated individuals is consistent with this hypothesis (Chung et al. 2002). If a familial aggregation of As methylation ability were to be found, it would suggest that genetic factors could underlie interindividual variation in As metabolism.

We noted a correlation of SMI values in the 11 pairs of children and their mothers that is consistent with a familial aggregation of As metabolic profile. Unfortunately, our small sample size makes this observation speculative until confirmed by a follow-up study of a larger sample size, which is currently being conducted. Similar differences in As metabolite concentrations and distribution of child–mother pair and child–adult, however, suggest that this finding is more systematic than random.

### Arsenicosis and exposure

Our study did not identify a single individual with arsenicosis even when the iAs exposure concentration was 160 μg/L, more than 3 times the 50-μg/L As concentration permitted by the Standards of Drinking Water Quality in China (Ministry of Health 2006). We suspect that this may be due to relatively short exposure time of 8–9 years. In China, individuals with arsenicosis have usually been exposed for more than 20 years (Liu et al. 2001), although we cannot rule out that better nutrition as a result of improved local economic conditions may play a role (Lammon and Hood 2004; Steinmaus et al. 2005).

### Limitation

Urinary As is recognized as a reliable indicator of recent exposure to iAs in drinking water and is often used as the bio-marker of As exposure (Calderon et al. 1999; Yamauchi and Fowler 1994). The concentrations of urinary As species in our study population increased with As exposure, reflecting iAs concentrations in the drinking water and thus can be used as a biomarker. Seafood is rare in the diet of the study population because of the poor economic conditions and the long distances of the villages from sea, thus we did not include this in our study. The unstable nature of trivalent forms of arsenicals (Aposhian et al. 2000; Del Razo et al. 2001; Gong et al. 2001; Le et al. 2000), especially MMA^III^, prevented us from evaluating the full spectrum of urinary As speciation. Our findings imply that children may benefit from having better secondary methylation than adults. Improved ability to quantify the unstable trivalent forms of As may hold the key in explaining the differences in SMI values between children and adults, as well as in people exposed to different levels of As. The correlation found in the methylation ability of child–mother pairs is interesting, but larger sample sizes and further investigation are needed.

## Figures and Tables

**Table 1:** Concentrations and percentages of As species in urine of children and adults.

	160-μg/L–exposed group GM (95% CI)		90-μg/L–exposed group GM (95% CI)		Control group GM (95% CI)	
	Children	Adults	Children	Adults	Children	Adults
*n*	44	56	47	50	16	20
Age, years	9.8 (9.0–10.6)	35.4 (32.6–38.1)	10.2 (9.3–11.1)	35.6 (32.5–38.6)	8.0 (6.4–8.9)	35 (31.1–38.1)
iAs (μg/g Cr)	82.4^a^,^c^,^e^ (63.9–106.2)	101.85^b^,^d^ (76.2–124.7)	48.7^a^,^d^ (39.2–60.5)	39.4^b^ (31.4–49.6)	1.0^b^ (0.7–1.7)	0.4 (0.3–0.5)
MMA (μg/g Cr)	70.8^a^,^c^,^e^ (57.4–87.3)	94.1^b^,^d^ (71.16–24.7)	41.1^a^,^d^ (34.7–48.6)	39.4^b^ (32.8–47.4)	2.9^b^ (2.3–3.9)	0.8 (0.6–1.1)
DMA (μg/g Cr)	408.8^a^,^c^,^e^ (329.6–507.0)	423.2^b^,^b^ (325.8–549.7)	206.1^a^,^d^ (172.4–246.3)	163.5^b^ (137.5–194.3)	29.3^b^ (22.0–39.0)	7.7 (5.4–11.1)
TAs (μg/g Cr)	573.5^a^,^c^,^e^ (464.8–707.5)	631.7^b^,^d^ (485.6–821.7)	304.8^a^,^d^ (257.4–361.1)	248.7^b^ (208.8–296.3)	34.5^b^ (26.5–45.0)	9.1 (6.5–12.7)
Percent iAs	12.7^a^,^c^ (10.9–14.6)	14.4^b^ (12.8–16.4)	16.0^a^ (14.1–18.3)	15.8^b^ (14.1–17.7)	2.8 (1.8–4.6)	4.16 (3.2–5.4)
Percent MMA	11.7^a^,^c^,^e^ (10.8–12.6)	14.2^b^ (13.1–15.3)	13.5a,^d^ (12.4–14.6)	15.8^b^ (14.6–17.0)	8.2 (7.0–9.6)	8.9 (7.0–11.2)
Percent DMA	73.3^a^,^c^,^e^ (71.1–75.6)	68.9^b^ (66.9–71.0)	67.6^a^,^d^ (64.5–70.7)	65.8^b^ (63.1–68.6)	84.6 (78.6–91.0)	85.0 (82.6–87.5)

Abbreviations: CI, confidence interval; Cr, creatinine; GM, geometric mean.

a *p* < 0.05, compared with children in 20-μg/L–exposed group.

b *p* < 0.05, compared with adults in 20-μg/L–exposed group.

c *p* < 0.05, compared with children in 90-μg/L–exposed group.

d *p* < 0.05, compared with adults in 90-μg/L–exposed group.

e *p* < 0.05, compared with adults in 160-μg/L–exposed group.

**Table 2:** Concentrations and percentages of urinary As species for 11 child–mother pairs in the 160 μg/L-exposed group.

	iAs [μg/g Cr (95% CI)]	MMA [μg/g Cr (95% CI)]	DMA [μg/g Cr (95% CI)]	TAs [μg/g Cr (95% CI)]	iAs [% (95%CI]	MMA [% (95%CI]	DMA [% (95%CI]
Mothers (GM)	30.8 (23.8–39.8)	34.9 (27.4–44.5)	172.8 (138.4–215.7)	241.0 (194.6–298.4)	12.8 (11.0–14.8)	14.5 (12.4–17.0)	71.7 (68.9–74.6)
Children (GM)	61.5 (45.6–82.9)	56.1 (38.9–80.8)	363.8 (300.4–440.4)	487.3 (391.9–606.1)	12.6 (11.2–14.2)	11.5 (9.2–14.4)	74.6 (71.4–78.0)
*p*-Value^a^	0.002	0.060	0.001	0.001	0.848	0.038	0.017

Abbreviations: CI, confidence interval; Cr, creatinine; GM, geometric mean.

a Differences between child–mother urinary iAs, MMA, DMA, and TAs were obtained by paired *t*-test.

**Table 3:** Concentrations and percentages (95% CI) of As species in urine for male and female.

	160-μg/L-exposed group		90-μg/L-exposed group	
	Male	Female	Male	Female
*n*	17	39	11	39
Age, years	30.1 (25.3–35.0)	37.7 (34.6–40.8)	35.0 (22.5–47.5)	31.4 (38.1–44.7)
iAs (μg/g Cr)	91.81 (56.80–148.40)	106.41 (74.13–183.93)	42.46 (27.54–65.47)	38.64 (29.52–50.58)
MMA (μg/g Cr)	86.73 (51.76–145.32)	97.57 (69.51–163.06)	41.71 (28.17–61.77)	38.80 (31.45–47.87)
DMA (μg/g Cr)	349.13 (213.72–570.35)	460.22 (337.88–734.91)	153.91 (95.17–248.90)	166.29 (139.01–198.93)
TAs (μg/g Cr)	537.27 (331.93–869.66)	677.88 (494.60–1092.75)	242.72 (155.00–380.07)	250.42 (207.28–302.55)
Percent iAs	15.92 (12.82–19.77)	13.84 (11.89–16.10)	17.25 (13.78–21.60)	15.40 (13.46–17.63)
Percent MMA	15.64 (14.18–17.25)	13.57 (12.31–14.97)	17.21 (14.52–20.38)	15.43 (14.18–16.79)
Percent DMA	66.30 (62.98–69.78)	70.10 (67.63–72.67)	63.50 (59.79–67.45)	66.43 (63.18–69.84)

Abbreviations: CI, confidence interval; Cr, creatinine; GM, geometric mean.

**Table 4:** Comparison of PMI and SMI and correlation analysis (95% CI) of methylation indices in 11 child-mother pairs.

				Correlation	
	Mother GM (95% CI)	Child GM (95% CI)	*p*-Value from paired *t*-test^a^	*r*	*p*-Value^b^
PMI	0.867 (0.849–0.887)	0.871 (0.854–0.888)	0.772	0.328	0.325
SMI	0.826 (0.799–0.855)	0.857 (0.822–0.893)	0.050	0.655	0.029

Abbreviations: CI, confidence interval; Cr, creatinine; GM, geometric mean.

a Significant difference in PMI or SMI between children and mothers.

b Significance of correlation in PMI or SMI between children and mothers.
